# Phylogenetic and Molecular Characterization of the Splicing Factor RBM4

**DOI:** 10.1371/journal.pone.0059092

**Published:** 2013-03-19

**Authors:** Chia-Chen Lu, Tz-Hao Chen, Jhe-Rong Wu, Hung-Hsi Chen, Hsin-Yi Yu, Woan-Yuh Tarn

**Affiliations:** Institute of Biomedical Sciences, Academia Sinica, Taipei, Taiwan; McGill University, Canada

## Abstract

The mammalian multi-functional RNA-binding motif 4 (RBM4) protein regulates alterative splicing of precursor mRNAs and thereby affects pancreas and muscle cell differentiation. RBM4 homologs exist in all metazoan lineages. The C-terminal unstructured domain of RBM4 is evolutionarily divergent and contains stretches of low-complexity sequences, including single amino acid and/or dipeptide repeats. Here we examined the splicing activity, phosphorylation potential, and subcellular localization of RBM4 homologs from a wide range of species. The results show that these RBM4 homologs exert different effects on 5′ splice site utilization and exon selection, and exhibit different subnuclear localization patterns. Therefore, the C-terminal domain of RBM4 may contribute to functional divergence between homologs. On the other hand, analysis of chimeric human RBM4 proteins containing heterologous sequences at the C-terminus revealed that the N-terminal RNA binding domain of RBM4 could have a dominant role in determining splicing outcome. Finally, all RBM4 homologs examined could be phosphorylated by an SR protein kinase, suggesting that they are regulated by a conserved mechanism in different species. This study offers a first clue to functional evolution of a splicing factor.

## Introduction

The RNA binding motif 4 (RBM4) protein and homologs are expressed in all metazoans. RBM4 contains two RNA recognition motifs (RRMs) and a CCHC-type zinc knuckle motif in the N-terminal region, and this region is highly conserved among species [1]. In contrast, its C-terminal domain has no discernible motif and is phylogenetically variable in sequence. Two RBM4 gene copies, namely *RBM4a* and *RBM4b*, in the human genome encode proteins with similar sequences, but their untranslated regions are largely divergent. The human RBM4 protein has multiple functions - primarily alternative splicing regulation and translational control [2], [3]. The C-terminal domain of human RBM4 contains a nuclear localization signal and also possesses nuclear export activity [1]. When this domain is fused with green fluorescence protein, it localizes the fusion protein to nuclear splicing factor-enriched speckles [1]. Moreover, the C-terminal domain confers the essential activity of directing 5′ splice site selection of the adenovirus early transcript, E1a.

Mammalian RBM4 proteins harbor three alanine-rich (Ala-rich) stretches in the C-terminal region. Polyalanine stretches composed of 7 to 20 alanines have been predicted in ∼500 human proteins, more than half of which are nuclear proteins [4]. Phylogenetic analyses have revealed that polyalanine coding sequences exist in gene families through convergent evolution and more frequently manifest among mammalian genes [5]. Expanded polyalanine tracts may cause protein misfolding and aggregation and may affect protein stability and cellular localization [4]. Polymorphism of polyalanine sequences may arise from genetic instability of repeated GCG trinucleotides [5]. Polyalanine expansion in roughly a dozen genes has been implicated in hereditary diseases [4]. Non-mammalian RBM4 homologs instead contain proline-rich tracts or arginine-serine (RS) dipeptides in the C-terminal domain (see Results). It is yet unclear why the C-terminal domain of RBM4 homologs has diverged and whether such evolutionary changes correlate with the function of RBM4 homologs.

*One of the major functions of mammalian RBM4 is to regulate* splicing of eukaryotic precursor mRNAs (pre-mRNAs), which is an important process for higher eukaryotic gene expression. Alternative splicing is essentially controlled by the interplay between RNA binding proteins and pre-mRNA *cis*-elements. RBM4 is capable of affecting 5′ splice site utilization and exon cassette selection [1]. RBM4 determines exon utilization through binding to CU-rich elements nearby the regulated exons [6], [7], [8], but its underlying mechanism still requires further investigation. It has been reported that *Drosophila* Lark, a mammalian RBM4 homolog, may regulate mRNA stability and thereby control circadian rhythm [9]. However, whether Lark also modulates pre-mRNA splicing has not been tested.

Although the function of human RBM4 has been essentially unveiled, less is known about non-mammalian RBM4 proteins. It is particularly interesting that the C-terminal domain of non-mammalian RBM4 homologs is largely divergent (see Results). In this study, we characterized RBM4 proteins from a wide variety of species to understand whether and how they may function in splicing regulation.

## Results

### Conservation and Divergence of RBM4 Proteins

**Figure 1:**
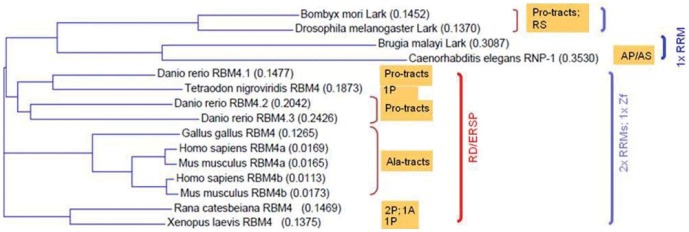
Phylogenetic analysis of RBM4 proteins. A phylogenetic tree of various metazoan RBM4 orthologs was created with the Clustal X program based on alignment of their full-length amino acid sequences. Branch lengths are drawn to scale, and weights for each RBM4 homolog sequence are given; a value of 0.01 represents a difference of 1% between two sequences. Each colored bracket indicates a group of species, in which RBM4 orthologs/homologs contain recognizable domains (RRM or zinc-finger; blue), the potential phosphorylation site RD/ERSP (red), or low-complexity sequences (brown). Detailed information for the low-complexity sequences is shown in Figure 2.

To better understand the structure-function relationship of RBM4, we first compared RBM4 homologs from representative species of metazoa. An evolutionary tree was generated by aligning the amino acid sequence of RBM4 homologs of species spanning *Caenorhabditis elegans* to human (Figure 1). Mammals and zebrafish (*Danio rerio*) have two and three RBM4 proteins, respectively; the duplicate or triplicate RBM4 genes are clustered on the same chromosome in each genome. However, only one RBM4 or putative RBM4 sequence of frogs (*Rana catesbeiana* and *Xenopus laevis*), chicken (*Gallus gallus*), and pufferfish (*Tetraodon nigroviridis*) could be directly retrieved or assembled from GenBank (see Materials and Methods for details). The homolog of vertebrate RBM4s in insects (*Drosophila melanogaster* and *Bombyx mori*), namely Lark, exists as only one copy per genome. *C. elegans* RNP-1 and *Brugia malayi* Lark have been designated as RBM4 homologs, but they are relatively divergent from all other RBM4 homologs (see below for details).

**Figure 2:**
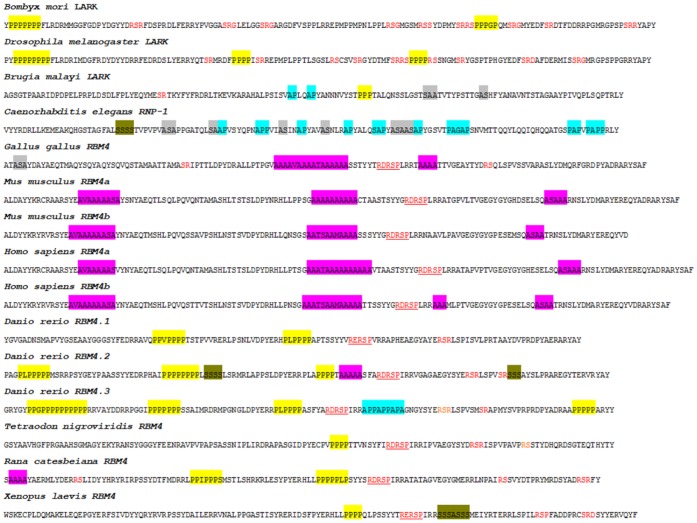
The C-terminal domain of RBM4 proteins. The C-terminal ∼150 residues of RBM4 homologs are shown. Colored boxes are low-complexity motifs including Ala-rich (pink), Pro-rich (yellow), Ser-rich (olive green), AP/PA (blue), and AS/SA (grey). RS dipeptides are highlighted in red; putative phosphorylation sites (RD/ERSP) are underlined.

Except for nematode RBM4-like proteins, all RBM4 homologs contain two RRMs and one zinc knuckle in the N-terminal half, of which the sequence is generally conserved between species-for example, human RBM4a and *Drosophila* Lark are still ∼50% identical in this region. In contrast, the C-terminal sequence of RBM4 homologs is divergent and contains one or more low-complexity motifs-for example, Ala-rich tracts in mammals and chickens, and Pro-rich sequences in fish and insects (Figure 2). The Ala-rich tracts are shorter than those of disease-associated polyalanine-containing proteins [4] and interrupted by other residues (Figure 2). Besides, the C-terminal domain of insect Lark proteins contains RS dipeptides, which are characteristic of many SR proteins [10]. Unlike typical SR proteins, however, the RS dipeptides of Lark are non-consecutive; therefore, whether Lark behaves as an SR splicing regulator has not been directly demonstrated. Nematode RBM4-like proteins are most divergent from RBM4s of other species; they contain only one RRM and no zinc finger, and particularly harbor different numbers of AP/PA and AS/SA dipeptides in the C-terminal domain.

Despite the low conservation of the C-terminal domain, the sequence RD/ERSP containing the major phosphorylation site (serine 309) of human RBM4 is essentially preserved in all vertebrates. Interestingly, S309 of human RBM4 can be phosphorylated *in vitro* by SR protein kinase [11]. We assumed that that SR protein kinase-mediated phosphorylation is preserved among RBM4 homologs including those of insects; we thus tested this hypothesis in our study.

### Drosophila Lark Functions as a Splicing Regulator

**Figure 3:**
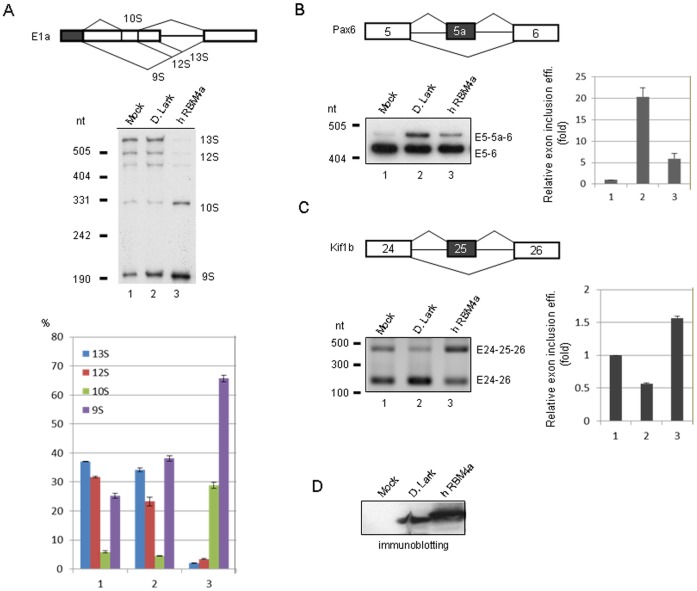
*Drosophila* Lark acts as a splicing regulatory protein. The expression vector of FLAG-tagged human RBM4a or *Drosophila* Lark was cotransfected with a splicing minigene reporter, adenovirus E1a (A), Pax6 (B) or Kif1b (C), into HeLa (A) or HEK293 (B and C) cells; the minigenes are shown in each panel. Mock represents the empty expression vector. To detect the splicing products, RT-PCR was performed using total RNA as template, and then the resulting products were separated on polyacrylamide gels (E1a and Pax6) or agarose gels (Kif1b). Except for Kif1b, RT-PCR products were further blotted onto membranes, and detected by hybridization with ^32^P-labeled specific probe (Table 1). For E1a, the bar graph shows the relative abundance (%) of the major splicing products. For Pax6 and Kif1b, relative exon inclusion efficiencies are indicated below the gel or blot. Exon inclusion efficiency was calculated as exon-included RNA/total RNA. Bar graph shows exon inclusion fold relative to the mock; bar graph shows average values with standard deviations obtained from three independent experiments. (D) Immunoblotting using anti-FLAG shows overexpressed FLAG-tagged Lark and RBM4a.

In this study, we attempted to compare the activity of RBM4 homologs by using the *in vivo* splicing assay. We co-transfected a splicing reporter and a vector expressing an RBM4 homolog into HeLa or HEK293 cells, and used reverse-transcription PCR (RT-PCR) to assess the splicing products. We previously showed that human RBM4a, but not *Drosophila* Lark, could activate the most distal 5′ splice site of the adenovirus E1a transcript [1]. However, in our recent experiments, we reproducibly observed that *Drosophila* Lark was able to induce 9S RNA expression in HeLa cells, albeit with a weaker activity than human RBM4a (Figure 3A). We assumed that this was due to improved expression of Lark (Figure 3D). Meanwhile, we fortuitously found that overexpression of *Drosophila* Lark could facilitate the inclusion of a rarely used alternative exon (exon 5a) of mouse Pax6 [12] in HEK293 cells, whereas human RBM4a had no effect (Figure 3B). Moreover, Lark and RBM4a exhibited an opposite effect on exon 25 selection of the mouse kinesin-like factor Kif1b transcript [13] (Figure 3C). Therefore, regardless of a lower expression level of Lark than RBM4a (Figure 3D), Lark could modulate not only 5′ splice site selection but also exon inclusion (*e.g.* Pax6) and exclusion (*e.g.* Kif1b), suggesting that Lark is a *bona fide* splicing regulator.

### Differential Activities of RBM4 Homologs in Alternative Splicing Regulation

**Figure 4:**
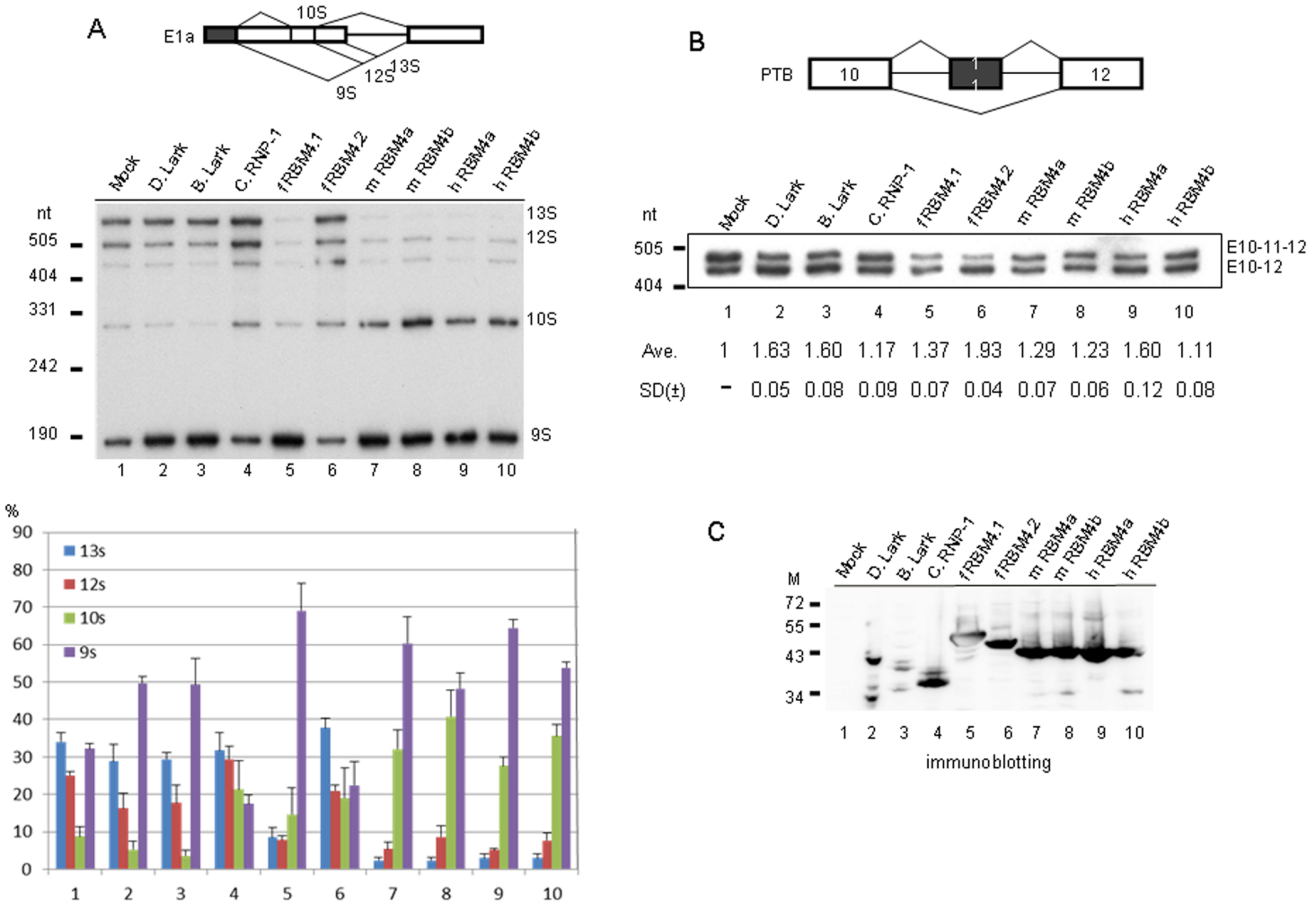
Evaluation of the alternative splicing activity of RBM4 homologs. *In vivo* splicing assay was performed using the E1a or PTB minigene reporter (diagrams). (A) HeLa cells were cotransfected with the E1a reporter vector along with the mock vector or vector expressing a FLAG-tagged RBM4 homolog. Alternative splicing products were detected and quantified as in Figure 3. (B) HeLa cells were cotransfected with the PTB reporter and an RBM4 homolog expression vector as in panel A. RT-PCR was performed using total RNA as template and specific primers to PTB (Table 1). Numbers below the blot indicate the relative exon skipping efficiency (exon-skipped RNA/total RNA) of each RBM4 transfectant vs. mock (lane 1; mock was set to 1); average and standard deviation were obtained from three independent experiments. (C) Immunoblotting using anti-FLAG shows overexpressed FLAG-tagged Lark and RBM4 proteins as indicated. M: protein molecular size markers (kDa). D, *Drosophila*; B, *Bombyx*; C, *Caenorhabditis*; f, fish; m, mouse; h, human.

Next, we evaluated the activity of RBM4 homologs from a wide variety of species using adenovirus E1a and mouse polypyrimidine tract binding protein (PTB) RNA as reporters. All mammalian RBM4 proteins and fish RBM4.1 more potently activated the distal 5′ splice site, yielding primarily 9S RNA with different levels of 10S RNA (Figure 4A, lanes 5,7–10). 10S RNA is produced by use of both 9S and 12S 5′ splice sites as well as a cryptic 3′ splice site between those two 5′ splice sites (Figure 4A, diagram). RBM4b reproducibly exhibited the strongest effect on activating 10S RNA expression (Figure 4A, lanes 8,10). In sharp contrast to RBM4.1, however, zebrafish RBM4.2 could not promote distal 5′ splice site usage (lane 6). As observed in Figure 3, *Drosophila* Lark could activate 9S RNA expression but exhibited a weaker activity than mammalian RBM4 and fish RBM4.1 (lane 2); a similar result was observed with *Bombyx* Lark (lane 3). The most divergent RBM4 ortholog *C. elegans* RNP-1 had no considerable activity (lane 4). Together, our data indicated that RBM4 paralogs act differently in 5′ splice site activation of E1a.

**Figure 5:**
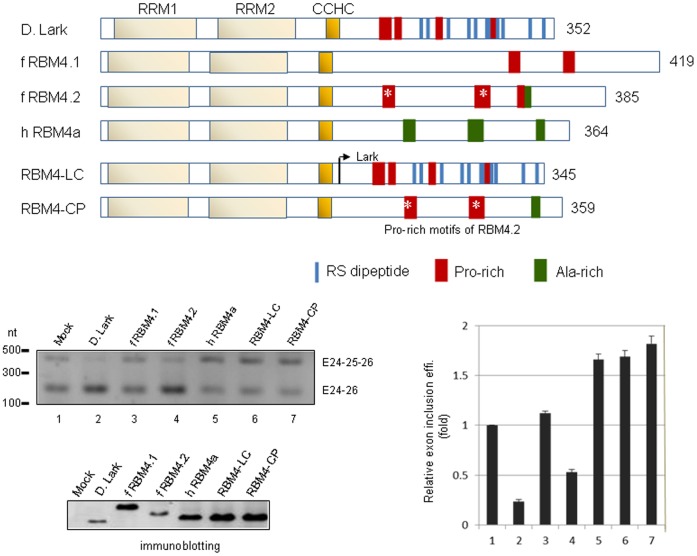
The N-terminal RNA binding domain of human RBM4 dominates the splicing effect. Diagram shows *Drosophila* Lark, zebrafish RBM4.1 and 4.2 and human RBM4a, and two chimeric proteins, RBM4-LC and RBM4-CP. RRM and CCHC represent RNA recognition motif and zinc knuckle, respectively. The *in vivo* splicing assay using the Kif1b reporter was performed as in Figure 3. Analysis of the splicing products was also as described in Figure 3. Relative efficiency for exon 25 inclusion was obtained from three independent experiments.

Human RBM4 promotes exon 11 skipping of the human PTB transcript via binding to the CU-rich elements within and nearby exon 11 [7]. Therefore, we established a PTB splicing reporter encompassing exons 9 to 11 of the mouse PTBP1 gene (equivalent to human PTBP1 exons 10–12) to examine the activity of RBM4 homologs/paralogs in exon utilization. In fact, mouse PTB exon 10 was partially included in HeLa cells (Figure 4B, lane 1), but was almost completely excluded from the reporter transcript in HEK293 cells (data not shown). Using HeLa cells, we observed that overexpression of human RBM4a induced exon 10 skipping in the PTB reporter (lane 9), consistent with the previous report that RBM4a promotes human PTB exon 11 skipping [7]. However, three other mammalian RBM4s had a relatively weaker activity than human RBM4a (lanes 7,8,10). More intriguingly, zebrafish RBM4.2 had a strong activity in inducing PTB exon 10 skipping, whereas RBM4.1 had only a minor effect (lanes 5,6). The difference between these two zebrafish RBM4s was further observed in the Kif1b exon selection (see Figure 5). *Drosophila* and *Bombyx* Larks were able to promote exon 11 skipping, regardless of their lowest expression level among all RBM4 homologs, whereas *C. elegans* RNP-1 could not (Figure 4B, lanes 2–4, and Figure 4C). Overall, our above splicing assays indicated that RBM4 homologs from evolutionarily distant species could act similarly (*e.g*. *Drosophila* Lark and mammalian RBM4 in alternative 5′ splice site usage of E1a and exon selection of PTB), whereas RBM4s of the same species (*e.g.* zebrafish) might exhibit different activities in splicing regulation (Figure 3, 4).

### The N-terminal RNA Binding Domain of Human RBM4 Dominates Splicing Decision

Using the Kif1b reporter, we observed that the RBM4 homologs of *Drosophila*, zebrafish and human exhibited different activities in exon 25 inclusion (see below for details). In light of their different C-terminal domain, we wondered whether this domain or even its simple amino acid repeats are sufficient to determine splice site selection. We made two chimeric human RBM4a proteins. RBM4-LC contained the N-terminal region of RBM4a and the *C*-terminal domain of *Drosophila L*ark, which has both Pro-rich motifs and RS dipeptides (Figure 5, diagram). In RBM4-CP, two *C*-terminal Ala-rich sequences of RBM4a were substituted by the *P*ro-rich sequences derived from zebrafish RBM4.2 (Figure 5, diagram). *In vivo* splicing of the Kif1b reporter transcript revealed that *Drosophila* Lark and human RBM4a promoted exon 25 exclusion and inclusion, respectively (Figure 5, lanes 2,5), consistent with our above observation (Figure 3). Zebrafish RBM4.2 acted as the same with *Drosophila* Lark whereas RBM4.1 had no effect (Figure 5, lanes 3,4). To our surprise, the two human RBM4a chimeras still promoted exon 25 inclusion (lanes 6,7). Therefore, neither the entire C-terminal domain of Lark nor the Ala-to-Pro substitutions could alter the splicing activity of human RBM4a in Kif1b exon selection, whereas the N-terminal RNA binding domain of human RBM4a might have a dominant effect on splicing regulation.

### Phosphorylation of RBM4 Homologs

**Figure 6:**
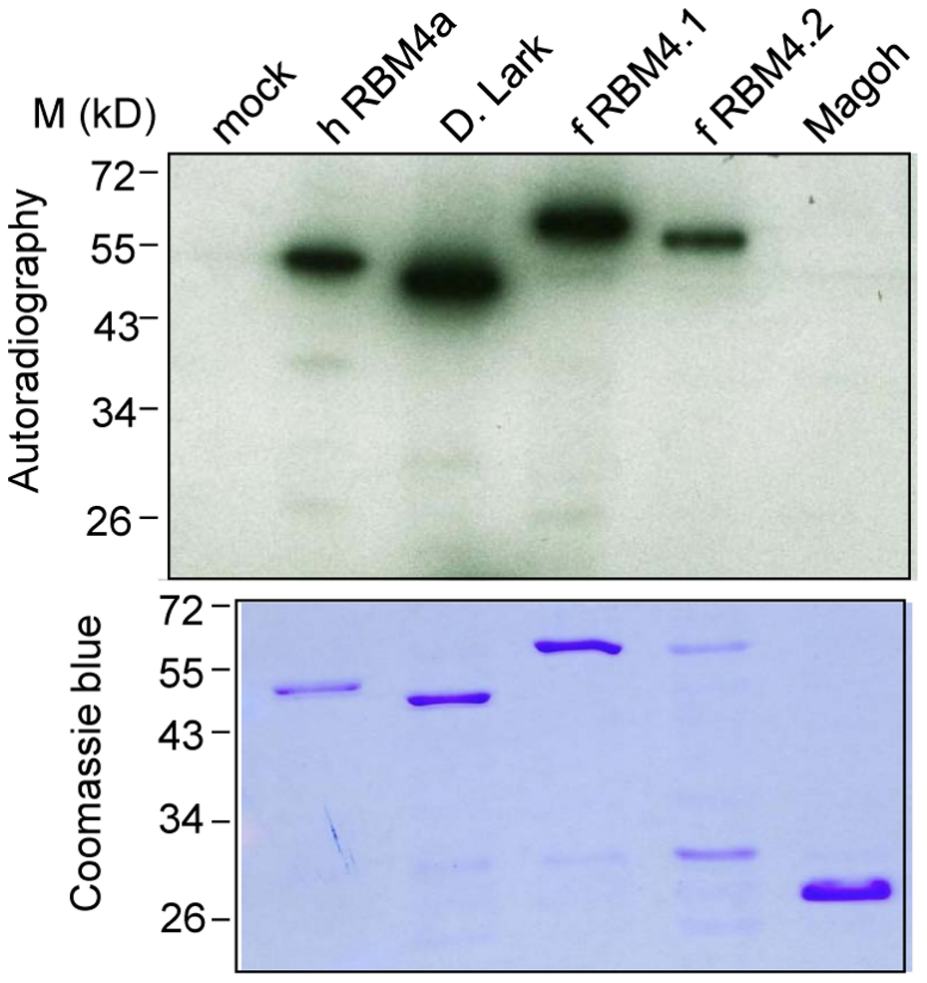
Phosphorylation of RBM4 homologs by SRPK1. *In vitro* phosphorylation of recombinant His-tagged RBM4 homologs and human Mago by GST-SRPK1 in the presence of [γ-^32^P]ATP. Proteins were fractionated by SDS-PAGE and detected by autoradiography (upper) and Coomassie blue staining (lower). Lane 1 shows the mock reaction. M: protein molecular size markers.

We previously reported that human RBM4a is phosphorylated in response to cell stress or differentiation [11]. Unlike SR splicing factors, RBM4 does not have repeated RS dipeptides, but its Ser309 can be phosphorylated by SR protein kinase *in vitro* [1]. The phosphorylation motif of RBM4, RDRSP, resembles that of yeast Npl3p [14]; both can be phosphorylated by mammalian SRPK1 [11], [14]. This motif is conserved among vertebrate RBM4 homologs except for an aspartate (D) to glutamate (E) change in a few species (Figure 2). Insect Lark lacks such a motif but contains several RS dipeptides. Therefore, we tested whether zebrafish RBM4.1 and RBM4.2 and *Drosophila* Lark could be phosphorylated by SRPK1. His-tagged RBM4 homologs were expressed in bacteria and affinity purified. The *in vitro* phosphorylation assay revealed that GST-SRPK1 could phosphorylate all recombinant RBM4 homologs but not the control protein, His-human Mago [15] (Figure 6). The RD/ERSP motif of zebrafish RBM4 proteins was likely phosphorylated because it fits well with the consensus of the SRPK1 target site [14]. Moreover, we conjectured that SRPK1 phosphorylated *Drosophila* Lark at RS(299)P, which is conserved between the two insect Larks examined. Thus, our data suggested that SRPK1-mediated phosphorylation of RBM4 homologs may also be conserved throughout evolution.

### Subcellular Localization of RBM4 Homologs

**Figure 7:**
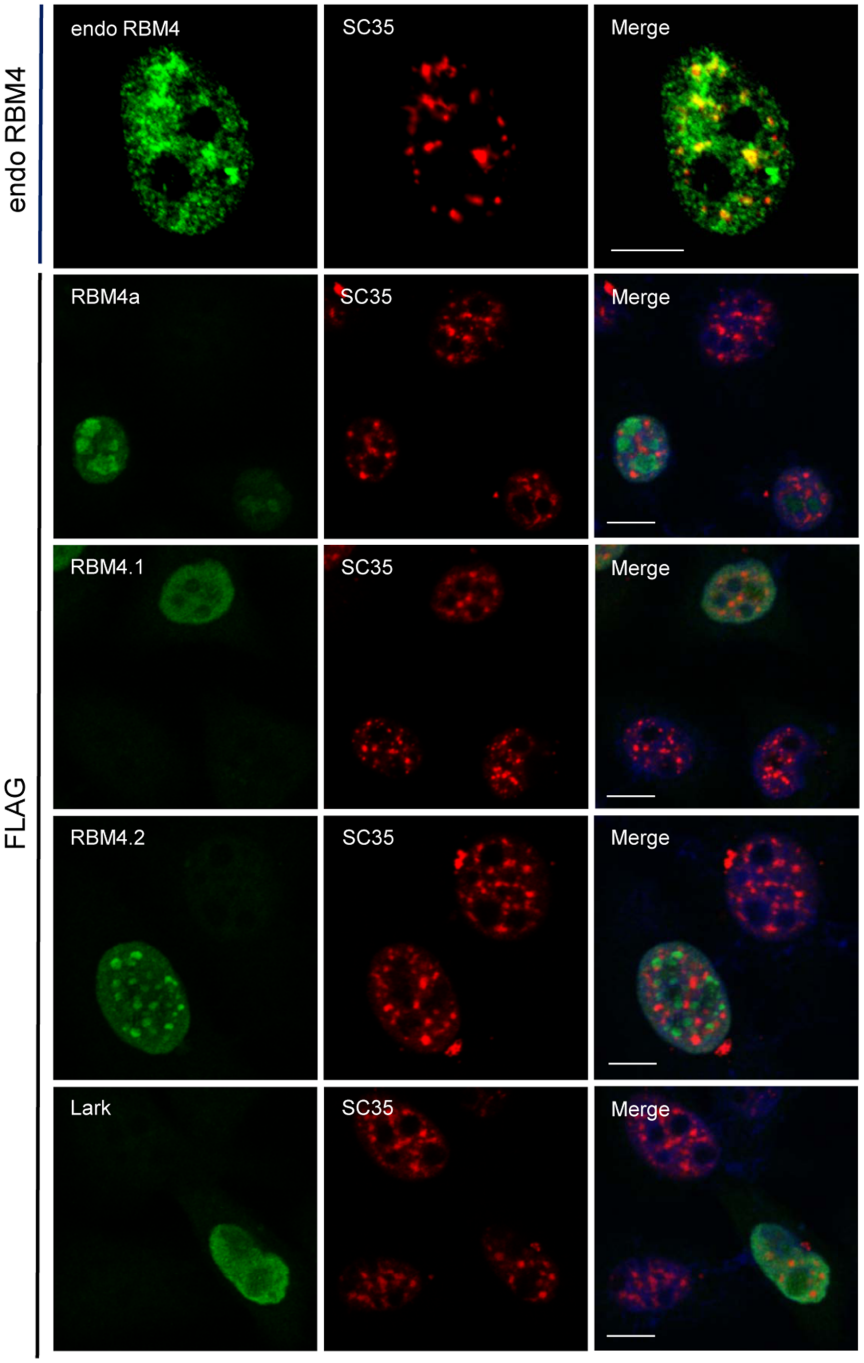
Cellular localization of RBM4 homologs. Top panels show endogenous (endo) RBM4: Double immunofluorescence was performed using anti-RBM4 and anti-SC35. To detect transiently expressed RBM4 orthologs, HeLa cells were transfected with a vector expressing FLAG-tagged human RBM4a, zebrafish RBM4.1 or RBM4.2, or *Drosophila* Lark, followed by immunofluorescence using anti-FLAG and anti-SC35. Cells were also stained with Hoechst 33258. Right panels show merged images. Scale bar, 8µm.

Finally, we examined the subcellular localization of RBM4 homologs in HeLa cells. Endogenous human RBM4 was distributed in the nucleoplasm and partially concentrated in splicing factor-rich speckles (Figure 7, endo RBM4), as previously reported [1], [16]. To compare RBM4 proteins of different species, we transiently overexpressed each in HeLa cells. Immunofluorescence showed that FLAG-tagged human RBM4a was predominantly located in nucleoli (Figure 7, FLAG-RBM4a), as evidenced by using anti-nucleolin (data not shown); this result was consistent with previous observations [1]. A similar result was observed for human RBM4b and two mouse RBM4 proteins (data not shown). However, transiently expressed zebrafish RBM4.1 and RBM4.2 showed very different localization patterns. RBM4.1 was distributed evenly throughout the nucleoplasm but excluded from nucleoli, whereas RBM4.2 localized to several large spots with irregular shapes in the nucleus, which did not overlap with splicing factor speckles (Figure 7). *Drosophila* Lark exhibited a diffuse nucleoplasmic distribution pattern (Figure 7), which was different from typical mammalian SR proteins [10]. Perhaps dispersed RS dipeptides of Lark are insufficient to direct its targeting to nuclear speckles.

## Discussion

The C-terminal domain of RBM4 mediates the protein’s interactions with other factors as well as a variety of RBM4 functions such as its intracellular transport, localization, and splicing regulation, and can be post-translationally modified in response to environmental and cellular cues [1], [2], [3], [6], [7], [8], [11], [17]. This study presents the first characterization of RBM4 orthologs and reveals variations in their splicing regulation and subcellular localization. Our results provide information on both the functional conservation and divergence of RBM4 homologs.

All RBM4 orthologs contain low-complexity amino acid motifs, of which the sequence has been changed throughout evolution. It has been reported that polyalanine domains in vertebrates are conserved between mammals but are rarer and shorter in chicken and zebrafish [5]. For example, the polyalanine tract exists in mammalian nuclear poly(A) binding protein (PABPN1) but is absent from its zebrafish homologs. Interestingly, both mammalian and chicken RBM4 proteins contain Ala-rich tracts but fish and frog RBM4s instead harbor Pro-rich sequences. This difference in the low-complexity amino acid motifs of RBM4 homologs reflects their functional diversification during evolution and/or between species. Moreover, the copy number of RBM4 genes ranges from one to three among species. Although the determination of whether duplicated RBM4 genes have any overlapping or specific function requires a knockout or transgenic animal study, our results suggest functional diversification of both RBM4 orthologs and paralogs.

As compared with polyglutamine expansion, our knowledge of the biochemical properties and functions of polyalanine motifs in proteins is relatively poor. Genetic disease-associated polyalanine expansion is found in a number of transcription factors as well as PABPN1 [4]. Polyalanine expansion may directly or indirectly influence the activity of transcription factors [4]. PABPN1 with an expanded polyalanine tract may sequester poly(A) RNAs in nuclear inclusions and causes global 3′ UTR shortening by activating proximal cleavage and polyadenylation sites of mRNAs [18], [19]. Mammalian RBM4 variants with slightly different lengths of Ala-rich tracts could be detected from cDNA libraries or in databases of expressed sequence tags (data not shown), but whether such a length polymorphism has any effect on RBM4 function remains to be examined. In contrast, polyprolines have been characterized in more detail. Pro-rich motifs can serve as a ligand for SH3 and WW domains [20]. Notably, the WW domain exists in a number of transcriptional co-activators and spliceosomal components, most of which have been implicated in splicing regulation [20]. Moreover, the preferential binding sites of the WW domain-containing yeast splicing factor PRP40 and its mammalian ortholog FBP11, *i.e.*, uninterrupted polyproline and PPLP (L denotes leucine) sequences, respectively, exist in Pro-rich RBM4 homologs. Perhaps these splicing factors and even some transcription factors engage in Pro-rich RBM4-mediated splicing regulation.

Our study first evaluated various RBM4 orthologs in alternative splicing of several mammalian transcripts, and showed that they differ considerably on splicing regulation (Figures 3, 4, 5). It is not surprising that *C. elegans* RNP-1 had almost no effect on splicing of the pre-mRNA reporters tested, because it is distantly related to RBM4 orthologs of other species. Intriguingly, zebrafish RBM4 paralogs exhibited grossly different splicing regulatory activity and cellular localization pattern (Figures 4,5,7). This result may reflect that duplicated RBM4 genes in an organism may have distinct biological function. On the other hand, distantly related RBM4 homologs may have similar functions; for example, both human RBM4a and *Drosophila* Lark could promote exon 11 skipping of PTB (Figure 4). Nevertheless, the C-terminal domain of *Drosophila* Lark is not sufficient to alter the splicing effect of human RBM4a on Kif1b exon selection (Figure 5), suggesting that the N-terminal RNA binding domain of RBM4a is responsible for splice site decision, at least in the cases examined. Perhaps RBM4 homologs bind somewhat different *cis*-elements, and thus determine the splicing outcome via a positional effect [21].

In conclusion, we provide the first line of evidence for both the conservation and divergence of RBM4 homologs in several biochemical and cell biology aspects. Our present data emphasize the potential role of RBM4s in splicing regulation, but it is also conceivable that they also participate in other aspects of RNA metabolism. Moreover, it is interesting to note that, regardless of their different sequences in the C-terminal domain, recombinant RBM4 homologs from *Drosophila* to human could be phosphorylated by SRPK1 (Figure 6), suggesting that RBM4s are regulated by an evolutionarily conserved mechanism. Nevertheless, a fuller understanding of the structure-function relationship of RBM4 homologs will require further investigation.

## Materials and Methods

### Plasmid Construction

The pcDNA3.1 (Invitrogen)-based vector for expressing FLAG-tagged human RBM4a was described previously [1]. Analogously, we constructed the FLAG-tag containing expression vectors for other RBM4 homologs. The cDNAs corresponding to human RBM4b and two mouse RBM4s were generated by RT-PCR using total RNA isolated from HeLa or C2C12 cells as template. The *Drosophila* Lark cDNA was derived from a previously described vector for expressing hemaglutinine-tagged Lark [1]. The cDNAs or cDNA libraries of *C. elegans*, *B. mori*, and zebrafish were obtained from Yi-Chun Wu (Taiwan University), Yu-Chan Chao and Bon-chu Chung (Academia Sinica), respectively. The chimeric RBM4-LC was made by fusing the N-terminal domain (residues 1–176) of human RBM4a with the C-terminal domain (residues 184–352) of *Drosophila* Lark. To construct the RBM4-CP expression vector, we sequentially used two sets of primers (D1F/D1R and D2F/D2R; see Table 1) to perform PCR on pcDNA3-FLAG-RBM4. The resulting truncated RBM4 lacked the regions coding for amino acids 233–240 and 281–299. Next, we inserted two Pro-rich sequences derived from zebrafish RBM4.2 into the above truncated RBM4 also using a PCR-based strategy. Two sets of primers for such insertions were RBM4.2 P1F/P1R and P2F/P2R (Table 1). The resulting RBM4-CP contained two Pro-rich sequences, PLPPPPPS and eight consecutive prolines. The E1a splicing reporter was described previously [1]. The Pax6 minigene construct was made by placing the PCR-amplified DNA fragment corresponding to exon 5 to exon 6 of mouse Pax6 (NT_039207.7) into pCH110. To construct the Kif1b minigene, three fragments corresponding to the mouse Kif1b genomic sequence (nt 85341–85767, 86609–87522, and 92413–92779 of NC_000070.5) were ligated and inserted into pCH110. The resulting construct contained the sequence of Kif1b exons 24–26 with truncated introns. The PTB minigene spanning exons 9–11 of the mouse PTBP1 gene (nt 21654959 to 21656016 of NT_039500.8) was also constructed in pCH110. The bacterial expression vectors encoding His-tagged RBM4 homologs including human RBM4a, zebrafish RBM4.1 and RBM4.2, and *Drosophila* Lark were constructed by inserting each cDNA into pET29b (Novagen) with appropriate restriction enzymes. The pET29b-hMago vector was used previously [15].

**Table 1:** PCR Primers.

Primer name	Sequence	Description
SV40 forward	TTTTGGAGGCCTAGGCTTTT	PCR probe for PTB and Pax6
E1a forward	GGTCTTGCAGGCTCCGGTTCTGGC	PCR
E1a reverse	GCAAGCTTGAGTGCCAGCGAGTAG	PCR probe for E1a
PTB reverse	CTGCCGTCTGCCATCTGCACAA	PCR
Pax6 reverse	TTGCCCTGGGTCTGATGGAG	PCR
Kif1b forward	ATGACAGCGAGACGACCA	PCR
Kif1b reverse	AATCCCCGGACTTCCCCC	PCR probe for Kif1b
RBM4 D1F	TCCGTGTATAATTACGCAGAGCAG	RBM4 Ala1 deletion
RBM4 D1R	CTCATAGGACCGGGCAGCACGG	RBM4 Ala1 deletion
RBM4 D2F	TCCACTTCATATTACGGGCGGG	RBM4 Ala2 deletion
RBM4 D2R	TCCTGAGGTGGGCAACAGGTG	RBM4 Ala2 deletion
F4.2 P1F	CCCCCACCCTCCGTGTATAATTACGCAGAGCA	Pro1 insertion
F4.2 P1R	AGGGGGAAGTGGCTCATAGGACCGGGCAGCAC	Pro1 insertion
F4.2 P2F	CCTCCTCCCCCCTCCACTTCATATTACGGGCG	Pro2 insertion
F4.2 P2R	TGGAGGAGGGGGTCCTGAGGTCGGCAACAGGT	Pro2 insertion

### Phylogenetic Analysis

The amino acid sequences of RBM4 homologs used for phylogenetic analysis were retrieved from GenBank (http://www.ncbi.nlm.nih.gov/genbank); their accession numbers are as follows: *D. melanogaster* Lark, NP_523957.1; *B. mori* Lark, NP_001037293.1; *C. elegans* RNP-1, NP_001256408; *B. malayi* Lark, XP_001902872.1; *R. catesbeiana* RBM4, ACO51643.1; *X. laevis* RBM4, NP_001087983.1; *D. rerio* RBM4.1, NP_955999.1, RBM4.2, NP_955971.1, and RBM4.3, NP_998482.1; *T. nigroviridis* RBM4, CAF93000.1; *G. gallus* RBM4, assembled from 1–242 of XP_003643577.1 and 247–372 of XP_003643963.1; mouse RBM4a, NP_033058.2 and RBM4b, NP_079993.2; human RBM4a, NP_002887.2 and RBM4b, NP_002887.2. We named *T. nigroviridis* CAF93000.1 RBM4 based on its apparent homology with other RBM4s and obtained a putative chicken RBM4 by assembling two overlapping, but perhaps incomplete, sequences in databases. A rooted phylogenetic tree based on amino acid sequence alignment was generated by the neighbor-joining method using Align X program (http://bioinformatics.uthsc.edu/classes/module5/alignments.html).

### In Vivo Splicing Assay

HEK293 or HeLa cells were cultured and transfected using Lipofectamine 2000 (Invitrogen) as described [1]. In general, 0.5 µg of a reporter plasmid was co-transfected with 2 µg of an effector expression vector into 7×10^5^ HEK 293 or 2.5×10^5^ HeLa cells for 24 h. Total RNA was collected from transfectants using Trizol reagent (Ambion). For RT-PCR, 2 µg of RNA was subjected to reverse transcription using SuperScript III (Invitrogen) and oligo(dT) as primer, and 1/20 of the reaction was analyzed by PCR using the primers listed in Table 1. Southern blotting and hybridization using ^32^P-labeled specific primers (Table 1) as probe were as described [1]. PCR bands were quantified by densitometry using ImageJ software (National Institutes of Health, USA).

### Immunoblotting and Immunofluorescence

Immunoblotting using an enhanced chemiluminescence reagent (Amersham) was carried out as described [1]; the primary antibodies used were polyclonal anti-FLAG (1∶2000 dilution; Sigma) and anti-hemagglutinin (anti-HA) (1∶2000 dilution; Bioman). The procedure for indirect immunofluorescence and Hoechst 33258 (Sigma) staining has been described [1]; the primary antibodies used included polyclonal anti-RBM4 (1∶100 dilution) [1], monoclonal anti-SC35 (1∶1000 dilution) and polyclonal anti-FLAG (1∶1000 dilution; Sigma). Samples were observed with a Carl Zeiss laser scanning confocal microscope (LSM510 META).

### In Vitro Phosphorylation of Recombinant Proteins

The C-terminally 6×His-tagged recombinant proteins were overexpressed in *Escherichia coli* strain BL21 by induction with 0.8 mM isopropyl-1-thio-β-d-galactopyranoside at 16°C for 16 h and then purified from bacterial lysates over nickel agarose (Novagen). Nickel agarose–bound proteins were eluted with 1 M imidazole and dialyzed against a buffer containing 20 mM HEPES (pH 7.9), 50 mM KCl, 0.2 mM EDTA, 1 mM dithiothreitol, and 20% glycerol. *In vitro* phosphorylation of RBM4 homologs was performed in a 20-µl reaction containing 0.4 µg of His-tagged RBM4 homolog proteins, 50 mM Tris-HCl (pH 7.4), 10 mM MgCl_2_, 1 mM dithiothreitol, 10 µM ATP, 0.2 µM [γ−^32^P]ATP, and 20 ng of GST-SRPK1 at 30°C for 1 h.
